# Effectiveness of gamified team competition as mHealth intervention for medical interns: a cluster micro-randomized trial

**DOI:** 10.1038/s41746-022-00746-y

**Published:** 2023-01-11

**Authors:** Jitao Wang, Yu Fang, Elena Frank, Maureen A. Walton, Margit Burmeister, Ambuj Tewari, Walter Dempsey, Timothy NeCamp, Srijan Sen, Zhenke Wu

**Affiliations:** 1 Department of Biostatistics, University of Michigan, Ann Arbor, MI USA; 2 Michigan Neuroscience Institute, University of Michigan, Ann Arbor, MI USA; 3 Department of Psychiatry, University of Michigan Medical School, Ann Arbor, MI USA; 4 Department of Computational Medicine and Bioinformatics, University of Michigan, Ann Arbor, MI USA; 5 Department of Statistics, University of Michigan, Ann Arbor, MI USA; 6 Institute of Social Research, University of Michigan, Ann Arbor, MI USA; 7 Data Bloom Consulting LLC, Cincinnati, OH USA

**Keywords:** Biostatistics, Human behaviour, Lifestyle modification

## Abstract

Gamification, the application of gaming elements to increase enjoyment and engagement, has the potential to improve the effectiveness of digital health interventions, while the effectiveness of competition gamification components remains poorly understood on residency. To address this gap, we evaluate the effect of smartphone-based gamified team competition intervention on daily step count and sleep duration via a micro-randomized trial on medical interns. Our aim is to assess potential improvements in the factors (namely step count and sleep) that may help interns cope with stress and improve well-being. In 1779 interns, team competition intervention significantly increases the mean daily step count by 105.8 steps (SE 35.8, *p* = 0.03) relative to the no competition arm, while does not significantly affect the mean daily sleep minutes (*p* = 0.76). Moderator analyses indicate that the causal effects of competition on daily step count and sleep minutes decreased by 14.5 steps (SE 10.2, *p* = 0.16) and 1.9 minutes (SE 0.6, *p* = 0.003) for each additional week-in-study, respectively. Intra-institutional competition negatively moderates the causal effect of competition upon daily step count by −90.3 steps (SE 86.5, *p* = 0.30). Our results show that gamified team competition delivered via mobile app significantly increases daily physical activity which suggests that team competition can function as a mobile health intervention tool to increase short-term physical activity levels for medical interns. Future improvements in strategies of forming competition opponents and introducing occasional competition breaks may improve the overall effectiveness.

## Introduction

Sufficient physical activity and sleep are associated with a lower risk for numerous health conditions, including cardiovascular disease, obesity, and depression^1–3^. However, only one in four U.S. adults meets the recommended 150 min of moderate-intensity activity per week^4^, and over one-third of U.S. adults do not achieve the recommended seven hours of sleep per night^5,6^. Medical internship, a one-year-long physician training program, is labeled short sleep and physical inactivity, which can lead to reduced health functioning and mental health symptoms^7^. Besides, the medical interns are often overwhelmed by a highly stressful working environment during residency, which makes conventional time-intensive intervention strategies (e.g., in-person workshops and face-to-face consultation) unavailable^8,9^.

Recent technical advances in wearable devices and mobile phones provide a new integrated platform to deliver interventions with minimal expense and user burden^10^ with the additional advantage of temporal and spatial flexibility. Mobile devices can collect real-time and objective measurements of a user’s physical activity and geographic location to provide personalized just-in-time adaptive interventions (JITAI)^11^. To date, many previous studies have shown the effectiveness of wearable and smartphone-based interventions on health outcomes^12–14^. Some studies included gamification, a strategy that attempts to enhance user enjoyment and engagement^15^ by introducing game mechanics into a non-game environment^16–18^. Theories of health behavior change suggest that gamification elements that prompt self-monitoring, such as performance feedback, progress monitoring, and social comparison have the potential to motivate changes in behavioral outcomes^19,20^.

Eight archetypes of gamification have emerged during the design of mHealth-related apps, and team competition is one such gamification strategy^21^. However, to our knowledge, its effectiveness on training residents at improving their health behaviors has not been formally assessed. There are two reasons why we started with team competitions. First, training physicians are known to be a particularly competitive population, and gamified competition has been shown to be effective among this group in other non-mobile contexts^22^. Second, given the stressful nature of internship year, we also hypothesize that a gamification strategy associated with increased fun and enjoyment might be most effective^23^.

Micro-randomized trials (MRT) can be used to address scientific questions about whether and under what circumstances JITAI components are effective, with the ultimate goal of developing effective and efficient JITAI^14,24,25^. In this study, we conduct a cluster MRT using principles of health behavior change and gamification to deliver a mobile app-based weekly team competition to evaluate the effectiveness of this type of mHealth intervention on individual physical activity, and sleep duration among a national cohort of first-year medical residents. We also explore the effectiveness of this mHealth intervention on user’s engagement and individual self-reported mood score, inspired by previous work showing that increased sleep opportunity and physical activity may improve an individual’s mood in this depression-vulnerable population^7,26^. In addition, we assess three potential effect moderators, variables that increase or decrease the effectiveness of mHealth intervention, to inform future research incorporating personalized team competition into mHealth intervention. Although we mainly focus on the effect of team competition on short-term (proximal) outcomes including step count and sleep minutes in this study, our ultimate goal is to improve medical interns’ long-term (distal) mental health and well-being by increasing their short-term physical activity and sleep duration.

## Results

### Study cohort

**Fig. 1:**
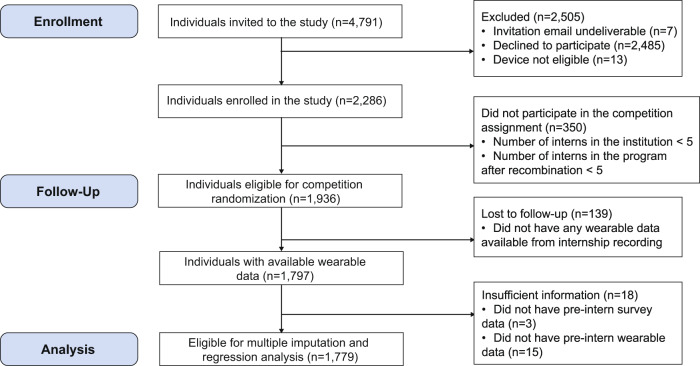
Study flow diagram. Flow diagram detailing the number of subjects who were enrolled, participated, randomized, and analyzed in the study.

**Fig. 2:**
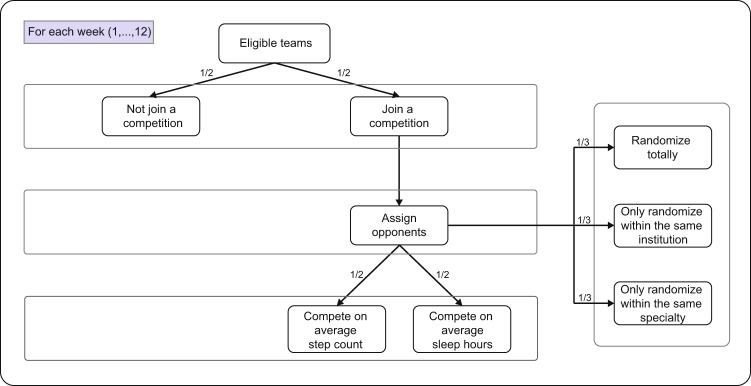
Study randomization scheme. Flow diagram demonstrating the randomization scheme of Intern Health micro-randomized trial.

Between 1 April 2020 and 16 June 2020, a total of 4791 incoming interns received the invitation email and 2286 (47.7%) interns enrolled in the study (see Fig. 1 for details of subject inclusion). Of those who enrolled, 84.7% (1936/2286) of participants could be grouped in a team with at least five interns, which were eligible for competition randomization, with a total of 191 teams. These eligible participants were randomized according to Fig. 2. Of the 1936 participants, 139 (7.2%) participants did not have any fitness tracker data available during the study, and 18 (0.9%) did not have sufficient pre-internship survey data and baseline data, which were excluded from the analysis. All remaining interns represented 90 residency institutions and 12 specialties. Among the 1779 (91.9%) participants included in the analysis, the mean age of the participants was 27.6 (SD 2.6). Males and females were nearly equally represented (54.5% female) (see Table 1 for more demographic information). Of the 1779 medical interns who were eligible for intervention, all of them were assigned to the competition arm at least once during the study and the mean number of weeks a participant was in the competition arm was 5.8 (SD 1.9) weeks.

**Table 1:** Demographics characteristics and specialty for study participants (*N* = 1779).

Demographic characteristics		Specialty	*N* (%)
Age (years), mean (SD)	27.6 (2.6)	Internal medicine	492 (27.7)
Sex (Female), *N* (%)	969 (54.5)	Surgery	240 (13.5)
Pre-internship baseline steps/day, mean (SD)	8121.0 (3228.9)	Pediatrics	215 (12.1)
Pre-internship baseline sleep mins/day, mean (SD)	420.6 (107.5)	Emergency medicine	145 (8.2)
Internship average days of competition, mean (SD)	40.7 (13.5)	Psychiatry	126 (7.1)
Race, *N* (%)		Ob/Gyn	104 (5.8)
White	957 (53.8)	Anesthesiology	94 (5.3)
Black/African American	106 (6.0)	Family medicine	78 (4.4)
Hispanic/Latino	96 (5.4)	Neurology	49 (2.8)
Asian	420 (23.6)	Med/Peds	38 (2.1)
Arab/Middle Eastern	31 (1.7)	Transitional	21 (1.2)
Other/Multiracial/Not reported	169 (9.4)	Other	177 (9.9)

*N* number of subjects, *SD* standard deviation.

### Main analysis

Main-effect analysis indicated that competing on step count had a significantly positive causal effect on proximal daily step count compared to the non-competition arm (see Table 2). The number of daily steps increased by 105.8 steps (SE 35.8, *p* = 0.03) for participants in the competition-step arm, compared to the non-competition arm. While no statistically significant effect on sleep duration was observed in response to competing on sleep. The estimate for the competition-sleep effect is −0.5 minutes (SE 1.7, *p* = 0.76).

**Table 2:** Parameter estimates for linear models assessing marginal and time-varying causal effect of team competition on daily step count and sleep duration.

		Main-effect analysis		Time-varying effect analysis	
Outcome & competition type	Parameter	Estimate	95% CI	Estimate	95% CI
Step	Intercept	7666.7	7546.0, 7787.3	7641.7	7514.9, 7768.6
	Week	−15.8	−24.7, −6.9	−11.0	−22.4, 0.4
	Competition	105.8	35.6, 176.0	185.3	51.6, 319.0
	Week: competition	–	–	−14.5	−34.6, 5.6
Sleep	Intercept	416.6	411.6, 421.1	413.2	408.3, 418.1
	Week	0.0	−0.3, 0.3	0.6	0.1, 1.0
	Competition	−0.5	−3.8, 2.8	9.8	2.9, 16.7
	Week: competition	–	–	−1.9	−3.1, −0.7

*CI* confidence interval.

### Moderation analysis

Moderation analyses were performed by adding linear interaction terms between effect moderator and intervention into the model (see Table 2). A negative though non-significant association was observed between the number of weeks in the study and competition-step intervention (−14.5 steps/day; SE 10.2; *p* = 0.16). That is, the moderation analysis (not the main-effect analysis) indicated that being in a competition-step week resulted in about 185.3 additional steps/day during the first week of the study, about 112.8 additional steps/day during the sixth week of the study, and about 25.8 additional steps/day during the twelfth week of the study. Similarly, a significantly negative interaction between the competition-sleep intervention and number of weeks in the study was identified: the causal effect of being in a competition-sleep week changed by −1.9 min/day (SE 0.6, *p* = 0.003) with each additional week in the study; note that at the beginning of the study (the first week), the causal effect was significantly positive 9.8 min/day (SE 3.4, *p* = 0.006), but then decreased. Plots of estimated causal effects of competition on proximal step count or sleep duration at different weeks and a sensitivity analysis to the linearity assumption (that the causal effect changes linearly by additional weeks in the study) were provided in Supplementary Fig. 3. We also assessed whether the causal effect of competition upon step count or sleep minutes (relative to no competition) would vary by the opponent team being from the same or a different institution or specialty (see Supplementary Table 4). Non-significant intra-institution negative moderation (−90.3 steps/day; SE 86.5; *p* = 0.30) and intra-specialty positive moderation (26.4 steps/day; SE 67.3; *p* = 0.69) of causal effect of competition on step were observed. Similarly, no statistically significant intra-institution (0.1 min/day; SE 2.7; *p* = 0.98) or intra-specialty moderation (−1.5 min/day; SE 2.7; *p* = 0.58) of causal effect of competition on sleep duration were observed.

### Exploratory analysis

Exploratory analyses assessed the causal effect of team competition on the proximal daily participation rates of step count and sleep minutes, averaged over all weeks (see Supplementary Table 5). The positive causal effect on daily participation rates of step count and sleep minutes was 0.3% (SE 0.2%, *p* = 0.14) and 0.9% (SE 0.2%, *p* = 0.0003) respectively. That is, if 1779 participants were all in the competition week, there would be additional 37 (1779 × 0.3% × 7) person-day records of step count and 112 (1779 × 0.9% × 7) person-day records of sleep minutes recorded within this week, compared to a non-competition week. We also assessed whether the causal effect of team competition had a positive impact on team-averaged mood score. Non-significant positive effect (0.02 units/day, SE 0.02, *p* = 0.25) of causal effect of team competition on mood score was observed (See Supplementary Table 6). Besides, we also assessed whether different device types (i.e., Apple Watch and Fitbit Charge) can moderate the effect of team competition and the results suggested that the main effect was not differed significantly by device types for both outcomes, daily step count and sleep minutes (see Supplementary Table 11). Similarly, we evaluated the heterogeneity of competition effect among different specialties and found that a significantly positive effect of team competition on step count was identified for Internal Medicine, Neurology, and Emergency Medicine, and a significantly negative effect of team competition on sleep minutes was recognized for Medicine & Pediatrics (See Supplementary Fig. 7).

## Discussion

This study answered two questions: 1. Is gamified team competition delivered via mobile app effective in improving health-related outcomes (daily step count and sleep minutes) of medical interns in the United States? 2. If it is effective, how to personalize and optimize the efficacy of team competition? The main-effect analysis indicated that the gamified team competition administered through smartphones can lead to increased proximal daily step count (105.8 steps/day). Positive causal effect suggested inclusion of competition via mobile app is a beneficial component of mHealth intervention. The moderator analysis demonstrated that week-in-study negatively moderates the efficacy of team competition, suggesting a waning causal effect of competition over time. Also, intra-institutional competition decreased the efficacy of competition, suggesting potential improvements in strategies of assigning opponent teams to boost the efficacy of team competition.

Xu et al. have identified eight randomized controlled trials reporting that mHealth-based gamification intervention exerted a positive impact on step count, and our finding on the beneficial effect of mobile-based gamified team competition upon physical activity is consistent with these previous studies^18,27–29^. For instance, Patel et al. found that mobile-based gamified competition can increase daily step count for overweight and obese adults in the United States^29^. However, the effect size from our study is smaller than previous studies^18,28–30^. There are multiple reasons. First, due to the highly stressful and intensive working environment, the medical interns may be less responsive to the intervention, compared to other less stressed populations. Second, the researchers in some previous studies tended to recruit participants who had already had some health issues. For example, Patel et al. recruited obese adults with BMI ≥ 25, aiming to use mobile-based gamification to promote physical activity^29^. Their intentions to be healthier (e.g., lose weight) could make them more willing to change behavior, thus leading to higher efficacy of the intervention. While the medical interns recruited in our study generally did not have special health conditions, thus the efficacy of team competition may not be boosted. In addition, our study provides a benchmark for future trials that seek to evaluate other competition-related app features that may improve the effectiveness of team competition, such as leaderboard, prize, and step/sleep goal achievement. Moreover, the strategy of how to pair opponents can also be optimized to increase the effect size. We consider the aforementioned content as the direction of future study.

One possible explanation for the waning causal effect of competition was that interns might be motivated when the study began and get tired later so they were less responsive to the competition assignment. The phenomenon of waning treatment effect is common in the field of mHealth intervention^13,31,32^ and further studies are needed to investigate how to extend the mHealth intervention effect. For example, a break could be given to the interns after an episode of a competition assignment to decrease their fatigue, and then intervention can be reintroduced to them after some rest time to regain the benefits from the intervention. Adding novel competition-related elements such as levels, scoreboard, and prizes could be another option.

One possible explanation for the negative impact of intra-institutional competition on the causal effect of competition on step count was that perhaps interns felt less competitive within their institution relative to extra-institutional members because they see their fellow institution members as colleagues. The negative moderation of intra-institutional competition suggested avoiding intra-institutional competition assignment in the future application to maximize the competition effect.

We also explored the causal effect of team competition on user’s engagement and mood score. A significant and positive effect of competition on the participation rate of sleep minutes was observed, indicating that the competition might have the potential to increase user’s engagement. In addition, a positive and non-significant causal effect of competition on mood score was observed. Besides, we also found that the device types did not moderate the effect of team competition, and the competition effect was modified by different specialties, indicating that identifying factors like specialty is worthwhile in the future study to avoid unplanned negative effects and boost the efficacy of the intervention.

Our study has multiple strengths. First, compared with standard single-time-point randomized controlled trial design, which can only inform moderation of causal effect by baseline variables (e.g., age, gender), micro-randomized trial enabled us to assess both causal effects of intervention components and time-varying moderation (e.g., week-in-study) of these effects. Second, a relatively large sample size (1779 participants in 191 teams) and long study period (12 weeks) allowed us to detect the causal effect of intervention, as well as effect moderators of interest. In fact, the data in our study has been collected for 26 weeks. However, we noticed that the percentage of missing data increased notably as the study went on, therefore, we chose to perform analysis on the first 12 weeks of the data. Third, the unique study population, medical interns with inherent hierarchical structure (by institutions and specialties), allowed us to assess the moderation of social connection and cooperation on the causal effect of gamified competition. Fourth, the analytical approaches we used in the study, the weighted and centered least squares estimator and multiple imputations, allowed us to assess the causal effect moderation consistently and robustly without requiring strong assumptions.

However, there are several unanswered questions and promising directions that should be addressed in future research. First, it remains unclear why competition did not affect participant’s sleep duration in the same way as step count. One of our conjectures is that the highly demanding working schedule during medical internship makes interns have little control over their sleep schedule, leading to insensitivity to competition intervention. Second, the reason that intra-institutional competition leads to negative moderation of causal effects of competition needs to be addressed in future study. Third, we have found that specialties may moderate the effect of competition. Future studies are warranted to investigate potential factors like specialty that can moderate the competition effect so that we can target individuals who can benefit most from the intervention. Fourth, we have found that the decreased participation rate of daily sleep minutes and self-survey mood score was positively related to losing previous competition, indicating that the engagement and competition performance of team members may be affected if the team lost the competition. Future research investigating the causal effect of competition result on competition performance and engagement is warranted. Fifth, in addition to competition, we are also interested in evaluating the effectiveness of other seven types of gamification approaches and hope to implement some of these other strategies via our mobile app in the future^21^.

Our study does have several limitations. The first is the data missingness and imputation. More than 30% data were missing for daily step count and 50% for daily sleep duration on individual level. Multiple imputation was used to impute the missing entries under the assumption of missing at random, however, the imputed values of a participant borrowed information from participants of other teams due to limited information in each team, which may result in attenuated estimate when assessing the moderation of competing within the same institution on causal effect of competition since the difference among teams can become smaller after imputation. Second, the results of this study may not extrapolate to a more general population because medical interns are different from the general population in terms of age, education level, and stress level. Individuals in the general population may be more responsive to the mHealth intervention than medical interns due to more flexible time. Therefore, to validate the generalizability of the results in and out of the training physician cohort, these suggested interventions should be further refined and replicated in additional studies and cohorts. Third, note that instead of individual-level analysis, cluster-level analysis, where each team was treated as the unit of analysis, was used to avoid ignoring the inherent clustering (team) structure using the team-level summary measures. The summary measures were calculated by taking the average of individuals’ measurements of the same team, which did not account for the heterogeneity among members within the team, resulting in reducing the power of the study. Further methodological research on statistical tools allowing analysis at the level of the individual while accounting for the clustering in the data in the field of MRT is needed.

In summary, through this smartphone-wearable-based prospective micro-randomized trial on training residents, we were able to identify the positive causal effect of team competition on proximal step count. The effect is considerably smaller relative to some prior studies, because we focused on factors associated with stress management (step count, sleep duration) in a population not actively seeking to lose weight or diseased. On the other hand, the team competition had no significant causal effect on sleep duration. Besides, we also found that the causal effects of competition were negatively moderated by week-in-study and intra-institutional competition. Exploratory analysis suggested that team competition may improve user’s engagement with the study app and the effectiveness of this intervention can be moderated by different specialties. These results suggest that gamified competition is worthy of inclusion in the mHealth intervention to improve the well-being of medical interns. Effectiveness of gamified team competition may be further boosted by introducing occasional breaks to mitigate waning effects over time, optimizing opponent assignment, or tailoring by specialty.

## Methods

### Study design and participants

We conducted a 3-month MRT to investigate the causal effects of team competition upon proximal weekly average daily step counts, minutes of sleep, participation rate, and mood score via the Intern+ mobile app as part of the Intern Health Study, a prospective cohort study assessing stress and depression during the first year of residency training in the Unitied States^33^. A convenience sample of training physicians, who began their internship in July 2020, were invited via email to participate in the study within one to three months prior to the start of internship. The lists of names and email addresses of eligible interns were either provided by the US healthcare institutions or gathered from publicly available medical school match lists. Ownership of an iPhone supporting iOS 10.0 or later or an Android device supporting version 6.0 or later was required. Upon enrollment, participants were provided with a Fitbit Charge 3 to collect sleep and activity data if they did not already own a compatible Fitbit or Apple Watch. All participants provided informed consent electronically and were compensated $80 to $130. The University of Michigan institutional review board approved the study. And the trial is registered with ClinicalTrials.gov, NCT05106439, 3 November 2021.

To protect participant anonymity, we required a minimum of five participating interns per team to be eligible for the competition arm of the study. Programs with at least five interns were grouped into program-based teams (e.g., “Michigan Psychiatry”). Interns within the same residency institution in programs that did not meet this criterion were grouped into institution-based teams (e.g., “Michigan Programs”), also with a minimum of five participants per team. All the remaining enrolled study subjects were considered ineligible for the competition arm.

All the eligible subjects were onboarded before their internships started on 1 July 2020. Baseline surveys assessed interns’ stress; also, baseline step counts, and sleep minutes were recorded via the Fitbits/Apple Watch. The competition assignment started on the first Monday following the start of internship (July 6, 2020) and ended on Sep 27, 2020 (Sunday of the 12th week), which lasted nearly three months. Each competition episode was one week, starting on Monday 00:00 and ending on Sunday 23:59.

### Randomization and masking

Each week, we repeatedly randomized interns by three factors: competition status (in competition or not), opponent team, and competition type (on step count or sleep minutes). Such a factorial design enables inference of causal effects of one or multiple factors based on the same study data. In particular, first, each team was randomized with equal probabilities to the competition or non-competition arm every Monday - this is the main randomization of the study. Second, every week, teams in the competition arm were randomly assigned an opponent team: (1) total randomization, where the opponent team was assigned regardless of institution and specialty (e.g., Michigan Pediatrics vs Yale Emergency Medicine); (2) intra-institutional randomization, where two competing teams were from the same institution (e.g., NYU Internal Medicine vs NYU Surgery); (3) intra-specialty randomization, where two opponent teams were from the same specialty (e.g., Northwestern Psychiatry vs OSU Psychiatry). All three rules for opponent assignment had equal probability (1/3) to be selected for each week. If there were an odd number of teams inside the randomization pool, then the team left over would be put back into the non-competition arm. Third, for each pair of opponent teams, there was a 50/50 chance of competing on average daily step counts or average daily sleep minutes. Figure 2 details the randomization scheme. Due to the nature of the intervention, participants could not be masked from the competition assignment. Although investigators were not masked to intervention allocation, all data collected from participants was through the app or wearable device.

### Procedures

After completing consent and downloading the study app, the wearable devices started to record daily step count and time spent asleep. Participants were prompted to report their daily mood (a score of 1 corresponded to the lowest and a score of 10 corresponded to the highest mood) every day at a user-specified time between 5 PM and 10 PM (default was 8 PM) in the study app. In addition to collecting data, the study app aggregated and displayed visual summaries of participant’s historical data, including daily step count, sleep minutes and mood score, through a dashboard which participants could access at any time via the app (see Supplementary Fig. 1). Separate from the competition component of the app, each user also had a 50/50 chance each day to receive a push notification at 3 pm which contained a message summarizing their personalized data feedback, a relevant fact or tip for improving mental health and well-being, or a general supportive statement. Furthermore, baseline and quarterly follow-up surveys were administered through the app.

The competition intervention was conducted for 12 weeks (Monday 6 July 2020, to Sunday 27 September 2020). Each team was randomly assigned to competition (intervention) and non-competition arm during each competition episode. Four competition-related smartphone push notifications were sent to participants in each competition week: (1) an alert of competition type (steps or sleep) and opponent team (Sunday 9 pm prior to the competition week), (2) two competition score updates (Wednesday 9 pm and Saturday 11 am during the competition week), and (3) the final competition results (Monday 12 pm following the competition week). Examples of messages are included in Supplementary Table 1. Participants could view their current competition scoreboard and competition history at any time via the Intern+ app. Supplementary Fig. 1 shows three representative screenshots of the app interface involving competition.

### Outcomes

The primary outcomes of the study were proximal weekly average daily step count and sleep minutes, by taking the average values of team members within a competition episode, which were measured by wearable devices (Fitbit or Apple Watch). The exploratory outcome was proximal weekly average daily participation rates of step count and sleep minutes, which was defined as the proportion of days that the participants in the team provided daily step count/sleep minutes within a competition week. The daily step count or sleep duration would be missing if the user was not wearing wearable devices during the daytime or nighttime. Demographic information and psychology-related scores were collected from baseline surveys (see details in Table 1).

### Missing data

Missing data occurred throughout the trial for various reasons: forgetting to wear a fitness tracker, only wearing fitness tracker during the day, technical glitches, and so on (see missingness information in Supplementary Fig. 2). Therefore, we used multiple imputation, a robust method for dealing with missing data, to impute the daily step count and minutes of sleep. For each day, the daily step count, sleep minutes, and mood score were imputed with predictor variables including step count, hours of sleep, and mood score from the previous three days, weekly average step count, sleep minutes, and mood score from the previous week and individual’s baseline characteristics including gender, depressive symptoms score (PHQ-9), neuroticism, early family environment. To accommodate the heterogeneity between different institutions and specialties, individual’s institution and specialties were added to the predictor variable list of imputation. R version 4.0.2 and *mice* function from R library *mice*^34^ were used to do the multiple imputation and predictive mean matching was selected as the imputation method. Results were pooled using 20 imputed datasets following Rubin’s rules^35^.

### Post-stratification weights

To reduce potential selection bias due to convenience sampling, we generated and applied post-stratification weights^36^ to account for the differences in demographics between enrolled subjects and the first-year interns nationwide in the 2020 cohort. We obtained the population data of first-year residents in the United States from the Association of American Medical Colleges (AAMC) in 2020. The data included the total number of residents and the number of surgical and nonsurgical residents from each cohort year. Within each specialty group from each cohort, we also obtained the numbers of women and men and numbers of White, Asian, and underrepresented minority residents. Using the data from the AAMC as the reference population, we did a 2-step post-stratification raking on Intern Health Study data to develop weights such that applying those weights results in a sample with a distribution of specialty, gender, and race that matches that of the AAMC data. The first step was to generate weights (w1) with the raking variables to be a specialty, and the second step was to generate weights (w1) with the raking variables to be gender and race within each specialty group (surgical and nonsurgical). And the final weights are the products of w1 and w2. The post-stratification rakings were performed with the R package “anesrake”^36^.

### Statistical analysis

Note that the competition assignment was randomized on the team level, therefore all the competition-related analyses in this paper were performed on the team level using summary measurements from each team, that is, the team was treated as the unit of analysis instead of an individual. Weekly team-based summary measurements were calculated by taking the average of individuals’ measurements within each team.

The primary aim of this study assessed whether there was a main causal effect of being in the competition arm on the team’s average proximal weekly average daily step count and sleep minutes, compared to not being in the competition arm. The primary analysis was done by fitting linear regression models using generalized estimating equation with independent working correlation matrix (R version 4.0.2; *geeglm* function from R library *geepack*) for average daily step count and sleep minutes separately, with competition assignment (i.e., intervention variable), number of weeks in study and control variables. Daily step count and sleep minutes were treated as continuous variables. The competition assignment variable was binary, with a value of 1 for being in a competition week and 0 for a non-competition week. The week-in-study was a continuous variable, with 0 for the first week of the study and 11 for the final week. Percentage of female, team average pre-intern measures, including daily step count, sleep minutes, psychology-related scores (e.g., PHQ-9 score), as well as team average previous week’s outcomes (i.e., previous week’s step count will be included if the outcome is current week’s step count), were included as control variables to increase the statistical power. The procedure implements a weighted and centered least square estimator (WCLS, details included in Supplementary Notes), proposed by Boruvka et al.^25^.

The secondary aims included moderation analyses to assess potential time-varying effect moderators, aimed at informing the design of real-time personalized and optimized delivery of mHealth intervention. Time-varying effect moderators are variables that can change the treatment effect and are time-varying because the values of moderators can vary across time (e.g., week-in-study)^24^. Two potential time-varying moderators of causal effect of competition were examined. The first moderation analysis is motivated by the hypothesis that the longer the participants were in the study, the more they may be accustomed to the competition intervention or become overburdened, leading them to become less responsive. Interaction terms between number of additional weeks in the study and the intervention variable were included in the model to evaluate the effect moderation. The second moderation analysis is motivated by the hypothesis that participants in the same institution or specialty tended to have stronger social connection, which may result in fiercer competition to boost the competition effect. Therefore, moderator analysis for whether intern was competing within the same institution or specialty was done by including two additional interaction terms between the intervention indicator and the intra-institution and intra-specialty indicators, respectively.

Exploratory analyses included assessing the causal effects of team competition upon user’s engagement and mood score averaged by team respectively. Linear probability model was used to assess the main causal effect of competition on proximal weekly average daily participation rates of step count and sleep minutes, motivated by the hypothesis that competition intervention can improve user’s engagement with the study app (see details in Supplementary Notes). The causal effect of team competition on team-averaged self-reported mood score was evaluated similarly to our analysis on step count (sleep minutes) in the main and secondary aims. Besides, we also examined two additional potential effect moderators, device types (Apple Watch and Fitbit Charge), and specialties. See details in Supplementary Notes.

All the analyses above were based on the weekly aggregated data because every week was treated as a complete episode of competition or not. For the moderation analyses, the results of these effects were reported from the models with linear moderators. The significance of regression coefficients for all analyses was tested through two-sided Wald test.

### Sensitivity analysis

To investigate the robustness of the results, we performed three types of sensitivity analysis. First, we compared the results from complete-case analysis and multiple imputations to evaluate the sensitivity of missing mechanisms. Second, we used a linear model for moderation in the main text, that is, the model for the treatment effect was specified as a linear function of the moderator. To assess the sensitivity of the linearity assumption, we explore potential non-linearity in the interaction term between the causal effect of competition and additional weeks in the study by replacing the linear function with a non-linear function *f*. Here we fit *f* using penalized basis spline by *gam* function from *mgcv* R package^37^ and natural cubic spline from *ns* function in R. The penalized basis spline models were fit using the restricted maximum likelihood method and thin plate regression spline as a smoothing basis. Third, to assess the sensitivity of different missingness patterns, the main results, which were from multiple imputation analysis, were compared with complete-case analysis after introducing a certain missingness pattern. Here we examined two different missingness patterns: dropout and weekly missingness. For dropout complete-case analysis, we removed imputed data from interns who dropped out of the study early. For example, if a user does not have any data points after 1 Sep 2020, all the imputed data points for this user after 1 Sep 2020, were removed. For weekly missingness complete-case analysis, we removed weeks with a large percentage of missing data in the outcome of interest. For example, we eliminated all weeks where more than five data points were missing before performing a complete-case analysis. The results of sensitivity analyses were detailed in Supplementary Notes.

## Supplementary information

Supplementary Information

## Data Availability

Deidentified data supporting the results and figures in this manuscript are available from the corresponding author upon reasonable request and completion of a data agreement with the Intern Health Study team.

## Code availability

Code for data preprocessing and statistical analysis will be made available online at https://github.com/jtwang95/IHS_competition.
