# Supplementary material for: Effectiveness of gamified team competition as mHealth intervention for medical interns: a cluster micro-randomized trial
Source: NPJ Digit Med. 2023 Jan 11;6:4. doi: 10.1038/s41746-022-00746-y (PMC9834206; doi:10.1038/s41746-022-00746-y)
Supplement: Supplementary file 1 — Supplementary Information [file 41746_2022_746_MOESM1_ESM.pdf]

Supplementary Materials

Supplement to: Effectiveness of gamified team competition as mHealth intervention for medical interns: a cluster micro-randomized trial

List of Supplementary Materials

- Page 3: Supplementary Notes
- Page 10: Supplementary Figures
- Page 17: Supplementary Tables
- Page 29: Supplementary References

Supplementary Notes

A weighted and centered least square estimator (WCLS)

To estimate the coefficients of interest with the existence of time-varying moderators, we used a weighted and centered least squares estimator proposed by Boruvka et al[2]. The method uses both the estimating equation method and robust "sandwich" estimate to provide consistent estimates and robust inference. The main advantage of the proposed method is that it does not require correct specifications to the terms that do not interact with treatment, in order to provide consistent estimates for the parameters of interest.

The method proposed by Boruvka et al. can provide valid inference for treatment-related variables when the treatment assignment probabilities are time-varying. Since the treatment assignment probabilities were constantly 0.25 across weeks in the IHS 2020 (each team had 50/50 chance to be in a competition week and for teams in competition week, they had 50/50 chance to compete on step count or sleep minutes), the weights in the estimating equation are all 1. In addition, the centering term for the treatment variable is always a constant 0.25.

The estimating equation method with robust error estimation has two main advantages: 1. It does not require distributional assumptions on continuous outcomes. 2. It allows dependence between observations in the data, where the observations for the same team in our dataset are correlated due to repeated measurements.

Models for assessing causal effect of competition on participation rate

To investigate the causal effect of competition on intern's participation rate on daily step count and sleep duration, a linear probability model was used to model the mean structure. More specifically, we modeled the probability that the daily step count or sleep duration was measured in week t as $E(Y_t|X_t, W_t, Z_t) = \beta_0 + \beta_1 Z_t + \beta_2 W_t + \beta_3 X_t$ for marginal-effect model, and $E(Y_t|X_t, W_t, Z_t) = \beta_0 + \beta_1 Z_t + \beta_2 W_t + \beta_3 Z_t W_t + \beta_4 X_t$ for time-varying-effect model, where Y_t is a continuous variable between 0 and 1 and the value of Y_t represents the proportion of available data points during week t for each team. Z_t is a binary treatment variable, where $Z_t = 1$ implies a competition week and $Z_t = 0$ implies a non-competition week. X_t is the set of control variables, including team-average baseline data measured before the weekly randomization, for the purpose of reducing variation in the outcome of interest Y_t. The set of variables X_t consists of percentage of female, team-average pre-intern daily step count, sleep minutes and psychology-related scores.

In the Intern Health Study data, whether the intern had a daily step count, sleep duration or self-reported mood survey is considered as a binary outcome. A conventional approach to model the binary outcome is to fit a logistic model. However, in our analysis, we did not adapt conventional logistic regression approach on daily data. Instead, we perform a weekly analysis using linear probability model and use a weighted and centered estimator proposed by Boruvka et al. to estimate the parameters of interest. The reason that we preferred a weekly analysis to a daily analysis is that the competition assignment was randomized on a weekly level and the daily level analysis violates the positivity assumption of the estimating method proposed by Boruvka et al[1]. Besides, the reason that we used linear probability model rather than beta regression model, which is more commonly used than linear probability model for proportional outcomes due to unit interval boundary, was that the theoretical guarantee of using beta regression to assess the time-varying causal effect moderation is not well-established. We calculated the predicted probability for each week in our dataset and all the estimated values fell between unit intervals, suggesting that the linear probability model is appropriate in our case.

Mood-score related analysis

Mental health disorders such as anxiety and depression are considered to be closely related to insufficient physical activity and sleep duration[2,3]. Therefore, we performed analyses on assessing the marginal and time-varying causal effect of competition on intern's mental health outcome: self-report mood score. We also did similar analyses on the participation rate of daily mood surveys. During mood-related analyses, the treatment variable competition was defined as either an intern was in competition step or sleep group. Here, we considered the competition indirectly affected the intern’s mood score since interns were competing on daily step count and sleep minutes, rather than directly on mood score.

The parameter estimates for linear models assessing the marginal and time-varying causal effect of competition on weekly average daily mood score were shown in Supplementary Table 6. From the marginal-effect model, we concluded that on average competition tended to improve the daily mood score with an estimated effect of 0.02 (SE 0.02, p=0.25). We also concluded that the additional weeks in the study was a significantly negative moderator of the causal effect of competition on daily mood score with an estimated moderation of -0.01 (SE 0.01, p=0.02) from the time-varying-effect model. The time-varying-effect plot showed that being in the competition arm had a significant positive effect on daily mood score at the early stage of the study and the effect waned over time.

The parameter estimates for linear models assessing the marginal and time-varying causal effect of competition on participation rate of daily mood survey were shown in Supplementary Table 5. Also, the estimated causal effect of competition on participation rate of daily mood survey at different weeks was shown in Supplementary Figure 4. We concluded that the competition did not affect the participation rate of daily mood surveys marginally. From Supplementary Figure 4, we can observe an interesting fact that the competition decreased the intern's participation rate of self-report mood surveys early in the study. Other than push notifications including life insight and tips received by all interns, the ones assigned to the competition arm received additional competition-related messages (see Supplementary Table 1) four times per week, which might make those less responsive to the push notifications and more possible to ignore the mood survey completion reminder 8:00 pm every night. Daily step and survey data were collected objectively through the fitness tracker, which was less sensitive to push notification fatigue. Intensive mHealth push notifications (overtreatment) may lead to inferior treatment effect; therefore, this gives rise to the need for just-in-time adaptive intervention (JITAI), which can deliver mHealth intervention optimally.

Device-specific moderation analysis

Previous studies have shown that Fitbit Charge 2 and Apple Watch 2 had similar accuracy in terms of estimating step counts[4,5], however, due to longer battery life, the Fitbit Charge series is more likely to be worn continuously, thus more likely to yield a higher step count and longer sleep duration in real life settings, compared with Apple Watch. To verify the existence of device-specific effect moderation on causal effect of team competition due to the heterogeneity of Apple Watch and Fitbit Charge (e.g., battery life, sensors), we performed additional moderation analyses by including an interaction term between intervention indicator and device type into the main-effect model.

The parameter estimates for linear models assessing the moderation of device types (Apple Watch and Fitbit Charge) on the effect of team competition on weekly average daily step count and sleep minutes were shown in Supplementary Table 11. The estimate of the interaction terms between device type and the indicator of team competition is -60.6 (SE 211.9, p=0.775) for step count and 0.3 (SE 7.3, p=0.971) for sleep minutes, which suggests that the heterogeneity of device types in terms of effect of team competition does not exist. Therefore, for the rest of the analysis, we did not perform separate analysis for Apple Watch and the Fitbit Charge.

Specialty-specific moderation analysis

We hypothesized that specialty might be a potential factor that can moderate the effect of team competition due to much variation in terms of call schedules among different specialties. For example, the need of 24/7 coverage means that the interns in Emergency Medicine usually work 12 or even 24-hour shifts, while the interns in Psychiatry may have more time flexibility. To verify the moderation of specialty on efficacy of competition, we performed moderation analyses by including an interaction term between competition indicator and specialty into the main-effect model.

The parameter estimates with 95% CI for linear models assessing the moderation of twelve different specialties on the effect of team competition on weekly average daily step count and sleep minutes were plotted in Supplementary Figure 7. A Significant and positive effect of team competition on daily step count was identified for Internal Medicine, Neurology and Transition. A Significant and negative effect of team competition on daily sleep minutes was identified for Medicine & Pediatrics. We concluded that the efficacy of team competition can be differed notably among different specialties, and specialty should be considered when administering team competition for medical interns.

Results of sensitivity analysis

Sensitivity of complete-case analysis

For primary aim, the estimate of the marginal causal effect of competition on step count from multiple imputation analysis was 105.8 (SE 35.8, p=0.003) steps, compared to 95.3 (SE 39.0, p=0.01) steps from complete-case analysis. The estimate of the causal effect of competition on sleep duration from multiple imputation analysis was -0.5 (SE 1.7, p=0.76) minutes, compared to 0.4 (SE 1.7, p=0.82) minutes from complete-case analysis. We can conclude that the conclusions for primary aim were mildly sensitive to missingness mechanisms.

For secondary aims, the estimate of the moderation of additional weeks in the study on the causal effect of competition on step count was -14.5 (SE 10.2, p=0.16) steps/week from multiple imputation analysis, compared to -13.3 (SE 10.8, p=0.22) steps/week from complete-case analysis. The estimate of the moderation of additional weeks in the study on the causal effect of competition on sleep duration was -1.9 (SE 0.6, p=0.003) minutes/week from multiple imputation analysis, compared to -1.1 (SE 0.5, p=0.03) minutes/week from complete-case analysis. The estimate of the moderation of competing within the same institution or specialty on the causal effect of competition on step count was -90.3 (SE 86.5, p=0.30) steps and 26.4 (SE 67.3, p=0.70) steps from multiple imputation analysis, compared to -132.6 (SE 92.6, p=0.15) steps and 35.7 (SE 69.5, p=0.26) steps from complete-case analysis. The estimate of the moderation of competing within the same institution or specialty on the causal effect of competition on sleep duration was 0.1 (SE

2.7, p=0.98) minutes and -1.5 (SE 2.7, p=0.58) minutes from multiple imputation analysis, compared to -2.4 (SE 3.1, p=0.43) minutes and 1.7 (SE 3.4, p=0.26) minutes from complete-case analysis. We can conclude that the conclusions for moderation of additional weeks in the study on causal effect of competition were insensitive to missingness mechanisms, while the conclusions for moderation of competing within the same institution or specialty on causal effect of competition were sensitive to missingness mechanisms. The size of the estimated moderation of competing within the same institution was enlarged when performing complete-case analysis and the sign of the moderation remained negative, matching the conclusions made in the main text.

For mood-related analysis, the estimate of the marginal causal effect of competition on mood score from multiple imputation analysis was 0.02 (SE 0.02, p=0.25), compared to -0.01 (SE 0.01, p=0.52) from complete-case analysis. The estimate of the moderation of additional weeks in the study on the causal effect of competition on mood score was -0.01 (SE 0.00, p=0.01) from multiple imputation analysis, compared to 0.04 (SE 0.03, p=0.14) from complete-case analysis. We can conclude that the conclusions for mood-related analysis were sensitive to missingness mechanisms.

The estimates of all the models mentioned above can be obtained through Supplementary Figure 2-4,6.

Sensitivity of non-linear moderation of treatment effect

The estimated causal effect of competition on step count or sleep duration at different weeks from nonlinear regression was plotted in Supplementary Figure 5. From the plots, we can see that linearity assumption is appropriate for our analysis.

The estimated causal effect of competition on participation rate of step count, sleep duration and mood survey at different weeks from nonlinear regression was plotted in Supplementary Figure 6. From the plots, we can notice some evidence of non-linearity, especially for step count. We observed that the efficacy decreased at first and then increased.

Sensitivity of missingness patterns

For primary aim, the estimate of marginal causal effect of competition on step count was 105.8 (SE 35.8, p=0.003) steps from multiple imputation analysis, compared to 104.1 (SE 38.6, p=0.007) steps from complete-case analysis with dropout and 100.4 (SE 38.4, p=0.009) steps from complete-case analysis with weekly missingness. The estimate of marginal causal effect of competition on sleep duration was -0.5 (SE 1.7, p=0.76) minutes from multiple imputation analysis, compared to 0.3 (SE 1.5, p=0.82) steps from complete-case analysis with dropout and 0.0 (SE 1.4, p=0.98)

minutes from complete-case analysis with weekly missingness. We concluded that the conclusions of the primary aim were robust to both dropout and weekly data missingness.

For secondary aims, the estimate of the moderation of additional weeks in the study on the causal effect of competition on step count was -14.5 (SE 10.2, p=0.16) steps/week from multiple imputation analysis, compared to -16.3 (SE 10.4, p=0.12) steps/week from complete-case analysis with dropout and -13.6 (SE 10.3, p=0.16) steps/week from complete-case analysis with weekly missingness. The estimate of the moderation of additional weeks in the study on the causal effect of competition on sleep duration was -1.9 (SE 0.6, p=0.003) minutes/week from multiple imputation analysis, compared to -1.5 (SE 0.5, p=0.004) minutes/week from complete-case analysis with dropout and -1.2 (SE 0.4, p=0.008) minutes/week from complete-case analysis with weekly missingness. The estimate of the moderation of competing within the same institution or specialty on the causal effect of competition on step count was -90.3 (SE 86.5, p=0.30) steps and 26.4 (SE 67.3, p=0.70) steps from multiple imputation analysis, compared to -100.4 (SE 89.5, p=0.26) steps and 27.8 (SE 70.2, p=0.69) steps from complete-case analysis with dropout and -111.1 (SE 92.9, p=0.23) steps and 22.3 (SE 71.2, p=0.75) steps from complete-case analysis with weekly missingness. The estimate of the moderation of competing within the same institution or specialty on the causal effect of competition on sleep duration was 0.1 (SE 2.7, p=0.98) minutes and -1.5 (SE 2.7, p=0.58) minutes from multiple imputation analysis, compared to -1.2 (SE 3.1, p=0.69) minutes and -0.8 (SE 2.9, p=0.78) minutes from complete-case analysis with dropout and -0.7 (SE 3.1, p=0.81) minutes and -0.4 (SE 2.9, p=0.88) minutes from complete-case analysis with weekly missingness. We can conclude that the conclusions for the effect of two moderators on the causal effect of competition were insensitive to dropout and weekly missingness, except that the moderation intra-institution competition is sensitive to the weekly missingness and dropout. The sign the moderation remained negative; however, the effect sizes were increased.

For mood-related analysis, the estimate of marginal causal effect of competition on mood score was 0.02 (SE 0.02, p=0.25) from multiple imputation analysis, compared to 0.03 (SE 0.02, p=0.14) from complete-case analysis with dropout and 0.03 (SE 0.02, p=0.15) from complete-case analysis with weekly missingness. The estimate of moderation of additional weeks in the study on causal effect of competition on mood score was -0.01 (SE 0.00, p=0.01) from multiple imputation analysis, compared to -0.02 (SE 0.01, p=0.01) from complete-case analysis with dropout and -0.02 (SE 0.01, p=0.006) from complete-case analysis with weekly missingness. We can conclude that the conclusions of mood-related analysis were insensitive to dropout and weekly missingness.

The estimates of all the models mentioned above can be obtained through Supplementary Table 7-10.

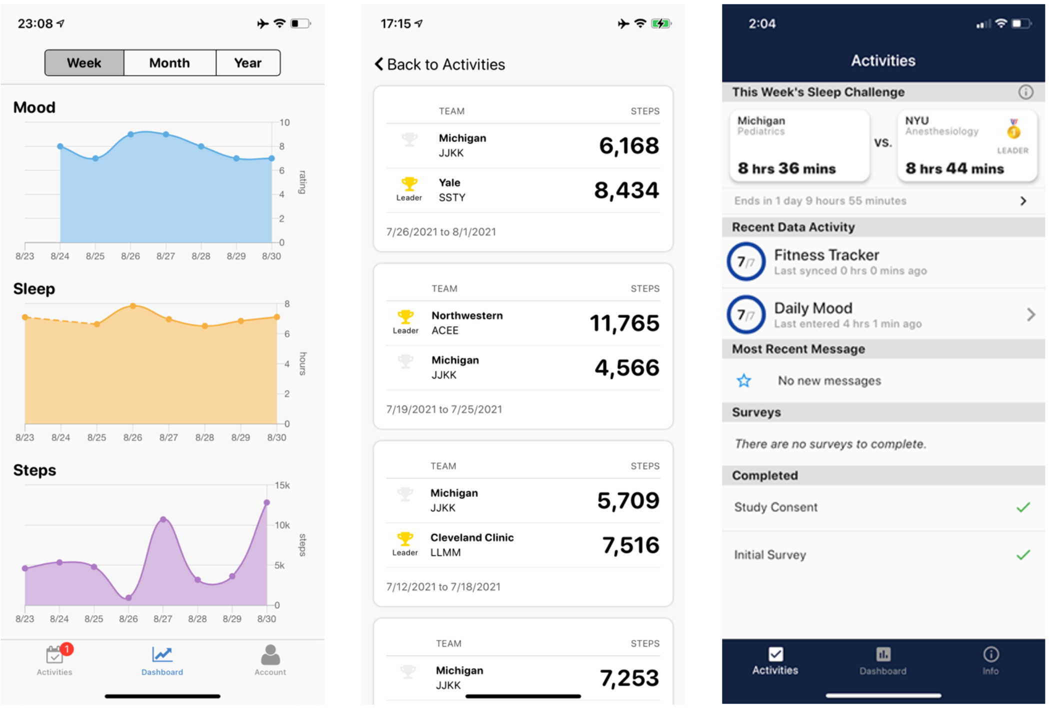

i) dashboard

ii) competition history

iii) competition assignment

Supplementary Figure 1: Screenshots of the app dashboard, previous competition history and weekly competition assignment. The screenshot of previous competition history contains pseudo program names.

[1](c) 2016-20222 The Regents of the University of Michigan, Intern + Mobile Application; Permission to use by Office of Innovation Partnership at University of Michigan.

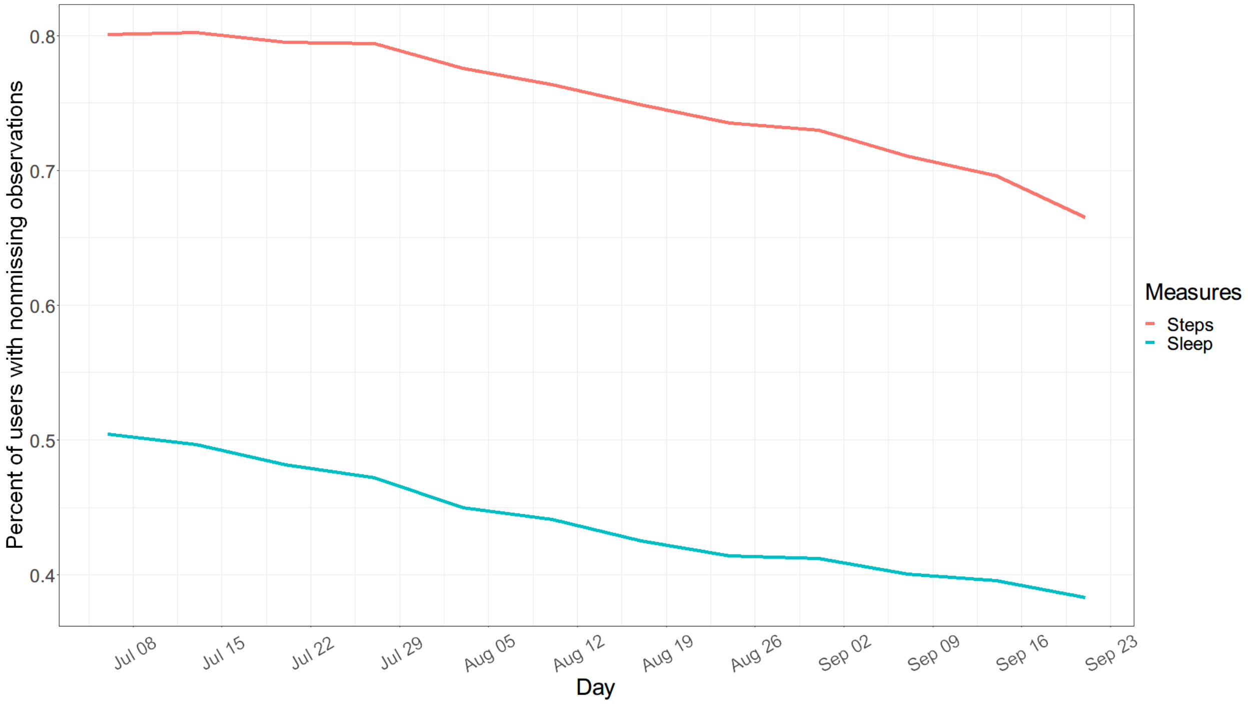

Supplementary Figure 2: Percentage of interns with nonmissing step and sleep observation for each day in the study. Red solid line indicates percentage of non-missing daily step count over time; Blue solid line indicates percentage of non-missing daily sleep record over time.

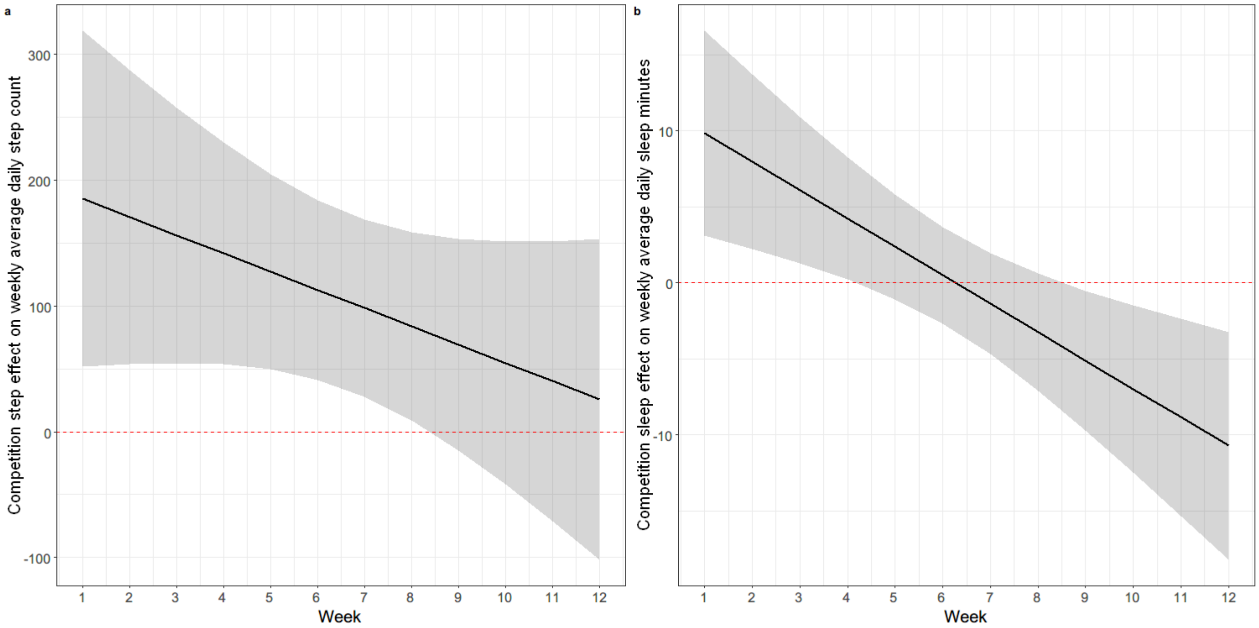

Supplementary Figure 3: a) Estimated causal effect of competition step on weekly average daily step count at different weeks. b) Estimated causal effect of competition sleep on weekly average daily sleep minutes at different weeks. Shaded area indicates 95% confidence interval. Red dotted line indicates no effect.

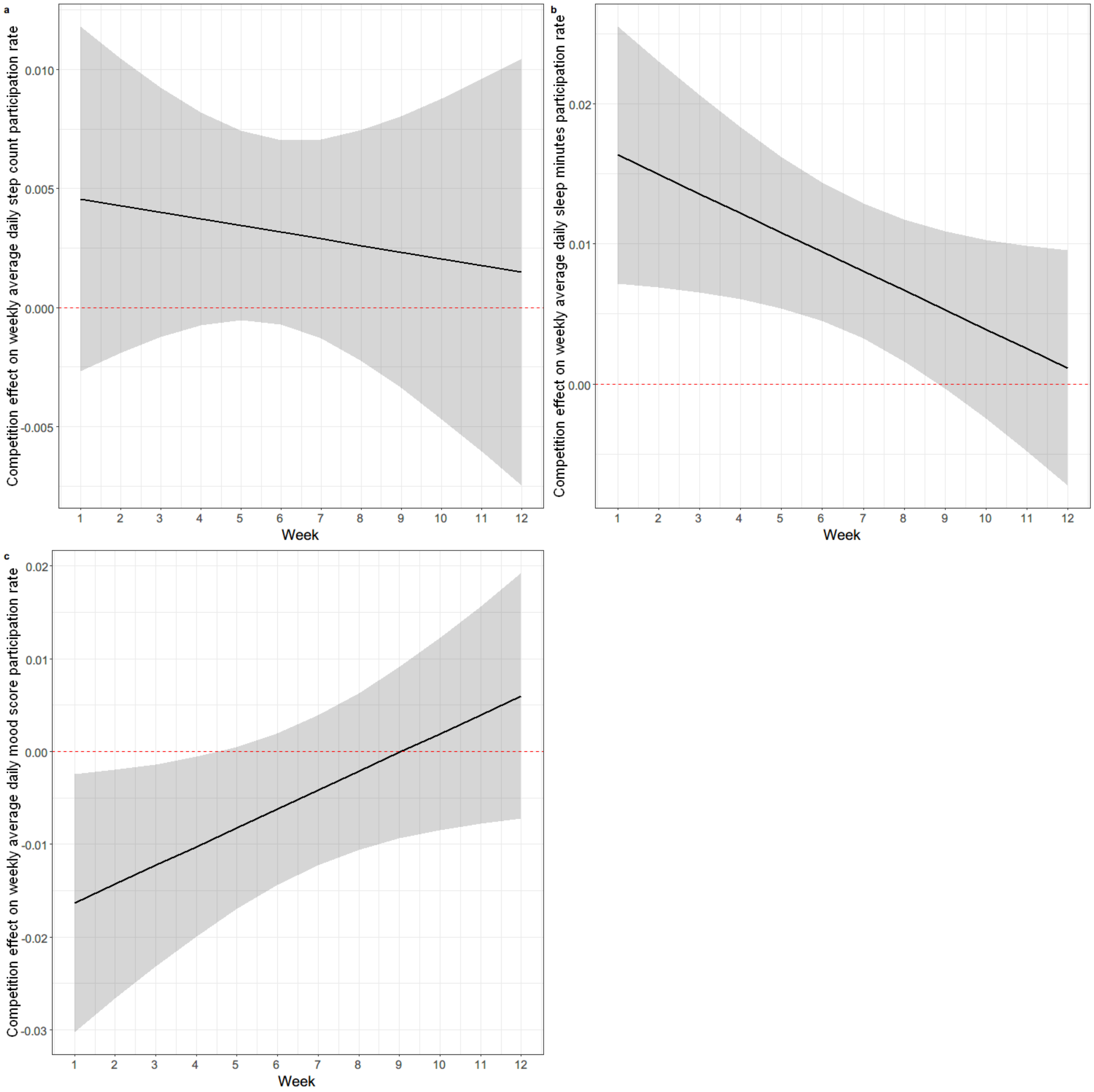

Supplementary Figure 4: Estimated causal effect of competition on the participation rate of a) daily step count, b) daily sleep minutes c) daily mood survey, at different weeks. Shaded area indicates 95% confidence interval. Red dotted line indicates the effect being 0.

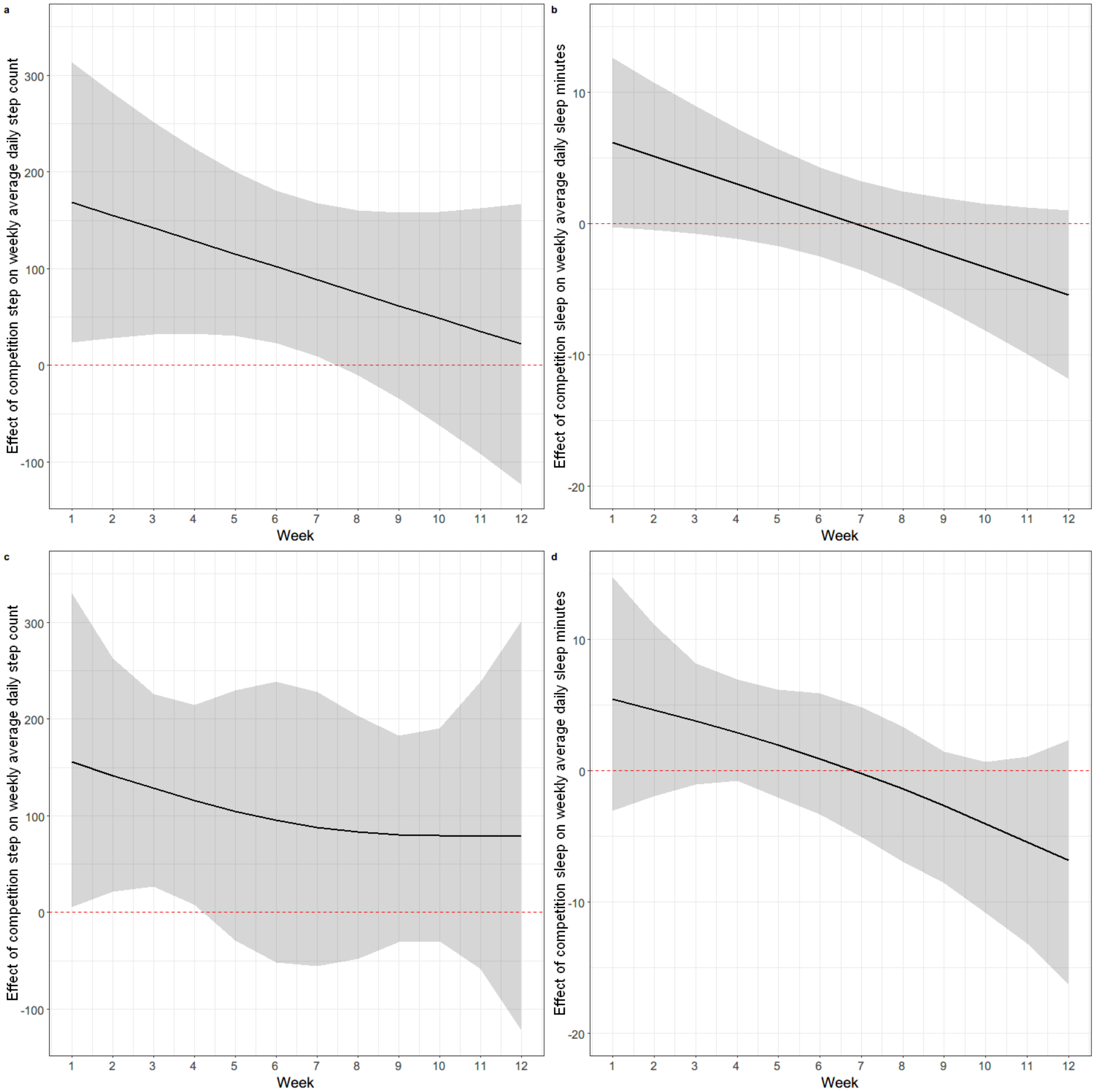

Supplementary Figure 5: a,b)Estimated causal effect of competition on weekly average daily a) step count and b) sleep duration at different weeks fitted using penalized basis spline. c,d) Estimated causal effect of competition on weekly average daily c) step count and d) sleep duration at different weeks fitted using natural cubic spline. Shaded area indicates 95% confidence interval. Red dotted line indicates no effect.

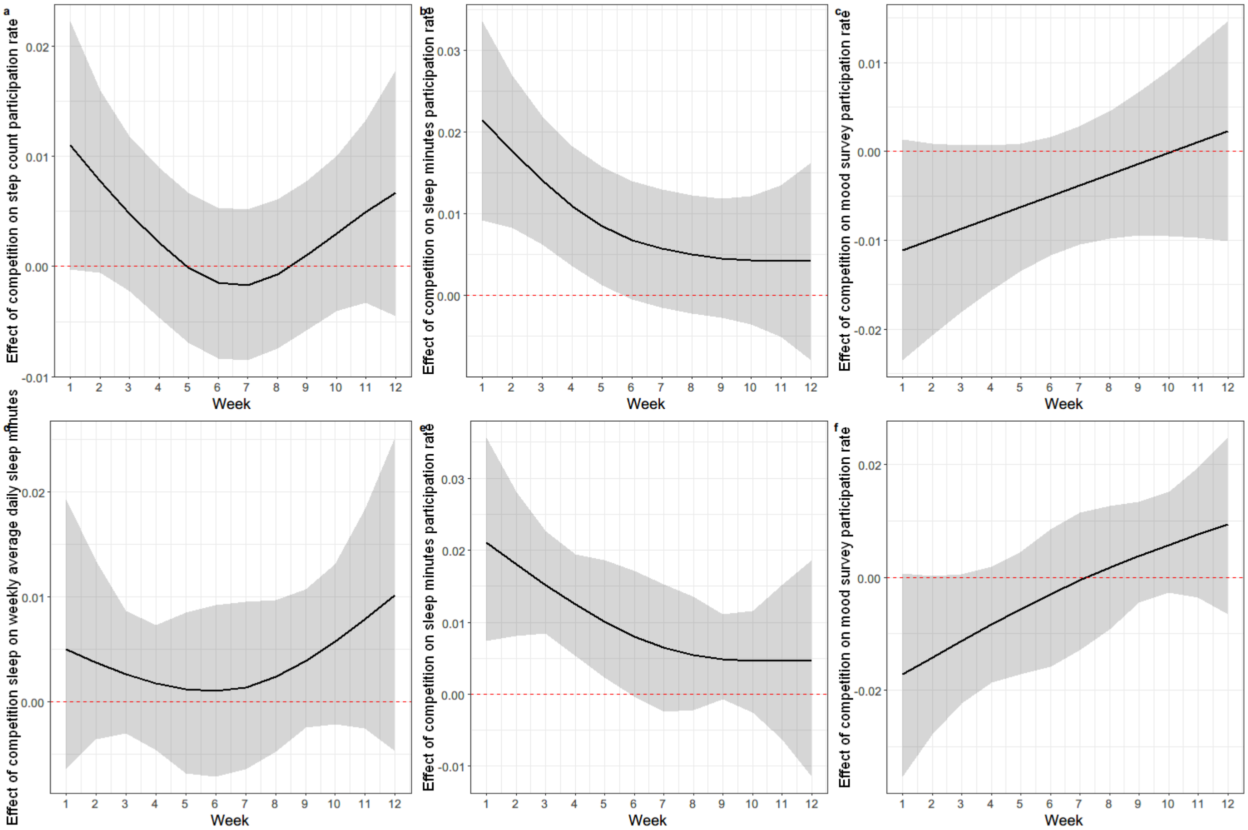

Supplementary Figure 6: a,b,c) Estimated causal effect of competition on participation rate of a) step count, b) sleep minutes, c) mood score at different weeks using penalized basis spline. d,e,f) Estimated causal effect of competition on participation rate of d) step count, e) sleep minutes, f) mood score at different weeks using natural cubic spline. Shaded area indicates 95% confidence interval. Red dotted line indicates no effect.

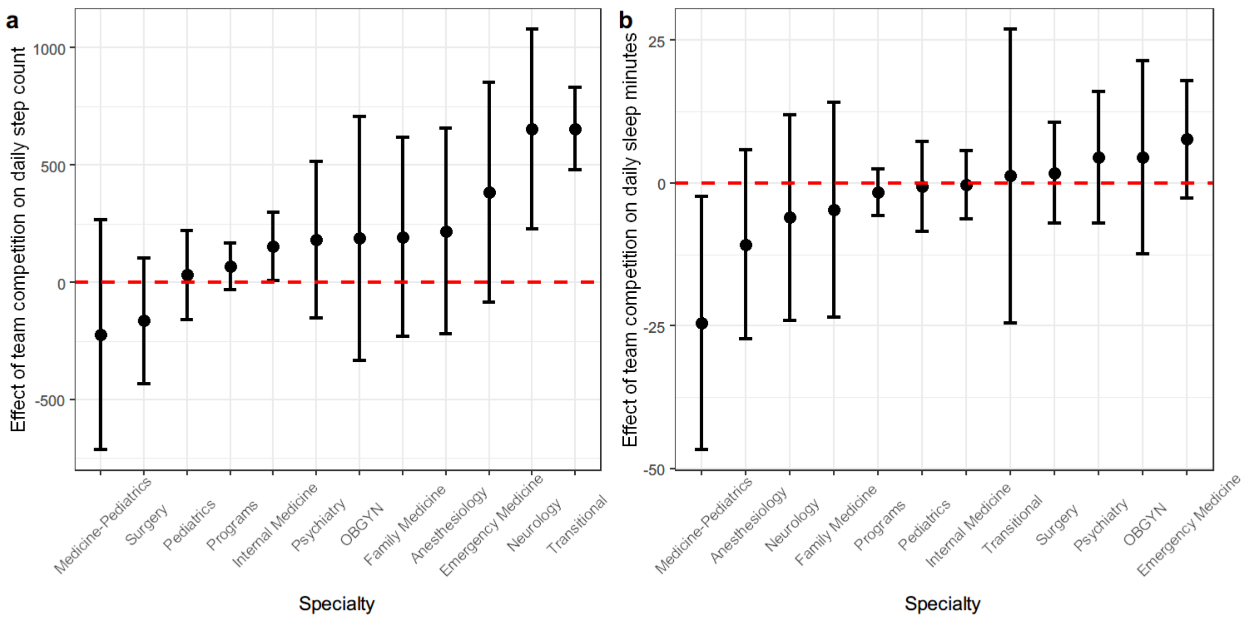

Supplementary Figure 7: a,b) Estimated marginal causal effect of competition on a) daily step count, b) daily sleep minutes, for twelve specialties, and the error bar indicates 95% confidence interval. Red dotted line indicates no effect.

Supplementary Table 1. Examples of different types of push notifications.

**Message types**	**Time**	**Examples**
Alert of competition types and opponent	Sunday 9:00 pm	MGH Surgery faces off against Northwestern Internal Medicine in this week's step competition!
Competition score status update	Wednesday 9:00 pm and Saturday 11:00 am	Yale Psychiatry is leading in this week's sleep challenge with an average of 8 hrs 41 min. Let's see who will win!
Competition final result	Monday 12:00 pm	Michigan Pediatrics comes out on top of this week's step challenge against NYU Anesthesiology. Great job to both teams!

Supplementary Table 2: Parameter estimates for linear model using complete-case and 20-time multiple imputation dataset, assessing marginal causal effect of competition on daily step count and sleep duration.

Outcome & Competition type		Complete case		Multiple Imputation	
	**Parameter**	**Estimate**	**95% CI**	**Estimate**	**95% CI**
**Step**	Intercept	7767.9	7648.7,7887.1	7666.7	7546.0,7787.3
	Week	-10.3	-18.9,-1.7	-15.8	-24.7,-6.9
	Competition Step	95.3	18.9,171.7	105.8	35.6, 176.0
**Sleep**	Intercept	414.5	408.6,420.4	416.6	411.6, 421.1
	Week	0.3	-0.1,0.7	0.0	-0.3, 0.3
	Competition Sleep	0.4	-2.9,3.6	-0.5	-3.8, 2.8

CI confidence interval.

Supplementary Table 3: Parameter estimates for linear model using complete-case and 20-time multiple imputation dataset, assessing time-varying causal effect of competition on daily step count and sleep duration.

Outcome & Competition type		Complete case		Multiple Imputation	
	Parameter	Estimate	95% CI	Estimate	95% CI
Step	Intercept	7744.9	7618.4, 7871.4	7641.7	7514.9,7768.6
	Week	-5.8	-18.1, 6.4	-11.0	-22.4, 0.4
	Competition Step	168.4	31.1, 305.8	185.3	51.6, 319.0
	Week: Competition Step	-13.3	-34.5, 7.8	-14.5	-34.6, 5.6
Sleep	Intercept	412.7	407.0, 418.5	413.2	408.3, 418.1
	Week	0.6	0.2, 1.1	0.6	0.1, 1.0
	Competition Sleep	6.2	1.0, 11.4	9.8	2.9, 16.7
	Week: Competition Sleep	-1.1	-2.0, -0.1	-1.9	-3.1, -0.7

CI confidence interval.

Supplementary Table 4: Parameter estimates for linear models using complete-case and 20-time multiple imputation dataset, assessing moderation of competing within the same institution on causal effect of competition on step count and sleep duration.

Outcome & Competition type		Complete Case		Multiple Imputation	
	Parameter	Estimate	95% CI	Estimate	95% CI
Step	Intercept	7745.3	7617.8, 7872.8	7641.9	7514.1, 7769.6
	Week	-5.8	-18.1, 6.4	-11.0	-22.4, 0.4
	Competition Step	186.1	38.4, 333.8	196.9	55.9, 337.9
	Week: Competition Step	-15.4	-36.6, 5.9	-15.9	-36.3, 4.5
	Competition Step : Same Institution Competition	-132.6	-314.2, 49.0	-90.3	-260.3, 79.8
	Competition Step : Same Specialty Competition	35.7	-100.4, 171.9	26.4	-105.9, 158.7
Sleep	Intercept	412.8	407.0, 418.6	413.2	408.3, 418.1
	Week	0.6	0.2, 1.1	0.6	0.1, 1.0
	Competition Sleep	6.6	0.6, 12.5	10.1	2.7, 17.5
	Week: Competition Sleep	-1.1	-2.1, -0.2	-1.8	-3.1, -0.6
	Competition Sleep : Same Institution Competition	-2.4	-8.6, 3.7	0.1	-5.2, 5.3
	Competition Sleep : Same Specialty Competition	1.7	-4.9, 8.3	-1.5	-6.9, 3.9

CI confidence interval.

Supplementary Table 5: Parameter estimates for linear models, assessing marginal and time-varying causal effect of competition on participation rate of daily step count, sleep duration and mood score(*100).

Model		Step		Sleep		Mood	
	Parameter	Estimate	95% CI	Estimate	95% CI	Estimate	95% CI
Main-effect	Intercept	76.8	76.1, 77.5	43.8	43.0, 44.5	51.4	49.9, 52.8
	Week	-0.3	-0.4, -0.3	-0.2	-0.2, -0.1	-0.6	-0.7, -0.5
	Competition	0.3	-0.1, 0.7	0.9	0.4, 1.4	-0.5	-1.3, 0.3
Time-varying-effect	Intercept	76.8	76.0, 77.5	43.4	42.6, 44.2	51.9	50.4, 53.4
	Week	-0.3	-0.4, -0.2	-0.1	-0.2, -0.0	-0.7	-0.9, -0.6
	Competition	0.5	-0.3, 1.2	1.6	0.7, 2.5	-1.6	-3.0, -0.2
	Week: Competition	-0.0	-0.2, 0.1	-0.1	-0.3, -0.0	0.2	0.0, 0.4

CI confidence interval.

Supplementary Table 6: Parameter estimates for linear model using complete-case and 20-time multiple imputation dataset, assessing marginal and time-varying causal effect of competition on mood score(*100).

Outcome & Competition type	Model	Parameter	Complete case		Multiple Imputation	
			Estimate	95% CI	Estimate	95% CI
Mood	Main-effect model	Intercept	719.0	713.8, 724.2	734.4	722.3, 746.6
		Week	-0.1	-0.5, 0.2	-1.6	-2.2, -1.0
		Competition	-1.0	-3.8, 1.9	2.1	-1.5, 5.8
	Time-varying-effect model	Intercept	716.9	711.3, 722.5	731.2	718.5, 743.8
		Week	0.3	-0.2, 0.8	-1.0	-1.8, -0.3
		Competition	3.7	-1.2, 8.6	9.0	2.3, 15.7
		Week: Competition	-0.8	-1.6, -0.0	-1.2	-2.3, -0.2

CI confidence interval.

Supplementary Table 7: Sensitivity analyses for assessing different missing patterns on marginal causal effect of competition on daily step count and sleep duration.

Missing pattern	Outcome & Competition type	Parameter	Estimate	95% CI
**Dropout**	**Step**	Intercept	7692.5	7579.9, 7805.1
		Week	-13.3	-21.8, -4.8
		Competition	104.1	28.3, 179.9
	**Sleep**	Intercept	415.3	411.2, 419.5
		Week	0.1	-0.3, 0.4
		Competition	0.3	-2.5, 3.2
**Weekly missingness**	**Step**	Intercept	7748.6	7639.4, 7857.8
		Week	-14.2	-22.5, -5.9
		Competition	100.4	25.0, 175.8
	**Sleep**	Intercept	415.4	411.3, 419.5
		Week	0.3	-0.0, 0.6
		Competition	0.0	-2.7, 2.7

CI confidence interval.

Supplementary Table 8: Sensitivity analyses for assessing different missing patterns on time-varying causal effect of competition on daily step count and sleep duration.

Missing pattern	Outcome & Competition type	Parameter	Estimate	95% CI
Dropout	Step	Intercept	7665.2	7547.4, 7783.1
		Week	-7.8	-19.3, 3.6
		Competition	189.7	51.7, 327.7
		Week: Competition	-16.3	-36.6, 4.1
	Sleep	Intercept	413.1	408.8, 417.4
		Week	0.5	0.1, 0.9
		Competition	7.8	2.2, 13.5
		Week: Competition	-1.5	-2.4, -0.5
Weekly missingness	Step	Intercept	7725.6	7611.1, 7840.1
		Week	-9.6	-20.9, 1.7
		Competition	172.4	33.7, 311.2
		Week: Competition	-13.6	-33.7, 6.5
	Sleep	Intercept	413.5	409.3, 417.8
		Week	0.6	0.3, 1.0
		Competition	6.3	1.1, 11.4
		Week: Competition	-1.2	-2.1, -0.3

CI confidence interval.

Supplementary Table 9: Sensitivity analyses for assessing different missing patterns on moderation of competing within the same institution or specialty on causal effect of competition on daily step count and sleep duration.

Missing pattern	Outcome & Competition type	Parameter	Estimate	95% CI
Dropout	Step	Intercept	7665.5	7546.7, 7784.3
		Week	-7.8	-19.3, 3.6
		Competition Step	201.2	54.7, 347.6
		Week: Competition Step	-17.5	-38.0, 3.1
		Competition Step : Same Institution Competition	-100.4	-276.1, 75.3
		Competition Step : Same Specialty Competition	27.8	-110.0, 165.6
	Sleep	Intercept	413.1	408.8, 417.5
		Week	0.5	0.1, 0.9
		Competition Sleep	8.3	2.2, 14.4
		Week: Competition Sleep	-1.5	-2.5, -0.5
		Competition Sleep : Same Institution Competition	-1.2	-7.2, 4.8
		Competition Sleep : Same Specialty Competition	-0.8	-6.6, 5.0
Weekly missingness	Step	Intercept	7726.2	7610.8, 7841.6
		Week	-9.6	-20.9, 1.7
		Competition Step	186.5	39.1, 333.8
		Week: Competition Step	-14.7	-35.0, 5.6
		Competition Step: Same Institution Competition	-111.1	-293.3, 71.0
		Competition Step: Same Specialty Competition	22.3	-117.4, 162.0
	Sleep	Intercept	413.5	409.3, 417.6
		Week	0.6	0.3, 1.0
		Competition Sleep	6.6	1.0, 12.1

		Competition Sleep : Same Institution Competition	-0.7	-6.9, 5.4
		Competition Sleep : Same Specialty Competition	-0.4	-6.1, 5.3

CI confidence interval.

Supplementary Table 10: Sensitivity analyses for assessing different missing patterns on time-varying causal effect of competition on causal effect of competition on daily mood score (*100).

Missing pattern	Model	Parameter	Estimate	95% CI
**Dropout**	**Main-effect model**	Intercept	731.2	718.3, 744.2
		Week	-1.8	-2.4, -1.1
		Competition	3.3	-1.1, 7.8
	**Time-varying-effect model**	Intercept	727.0	713.9, 740.2
		Week	-0.9	-1.9, 0.0
		Competition	12.1	4.1, 20.2
		Week: Competition	-1.7	-3.0, -0.4
**Weekly missingness**	**Main-effect model**	Intercept	736.5	724.5, 748.5
		Week	-1.6	-2.2, -1.0
		Competition	3.2	-1.1, 7.5
	**Time-varying-effect model**	Intercept	732.4	720.0, 744.8
		Week	-0.8	-1.6, -0.0
		Competition	11.5	3.9, 19.1
		Week: Competition	-1.7	-2.9, -0.4

CI confidence interval.

Supplementary Table 11: Parameter estimates for linear models using complete-case and 20-time multiple imputation dataset, assessing moderation of device types on causal effect of competition on step count and sleep duration.

Outcome & Competition type		Complete Case		Multiple Imputation	
	Parameter	Estimate	95% CI	Estimate	95% CI
Step	Intercept	7774.8	7584.8, 7964.8	7714.4	7543.7, 7885.2
	Week	-12.6	-23.0, -2.3	-15.8	-24.7, -6.9
	Competition Step	205.6	-120.9, 433.0	131.6	-51.3, 314.4
	Competition Step : Apple Watch	-236.9	-771.6, 297.8	-60.6	-477.5, 356.2
Sleep	Intercept	411.1	400.9, 421.2	411.8	405.1, 418.5
	Week	0.3	-0.1, 0.8	0.0	-0.3, 0.4
	Competition Sleep	-3.5	-12.3, 5.3	-0.6	-7.2, 6.1
	Competition Sleep : Apple Watch	7.7	-15.5, 30.9	0.3	-14.3, 14.8

CI confidence interval.

Supplementary References

1. Boruvka, A., Almirall, D., Witkiewitz, K. & Murphy, S. A. Assessing Time-Varying Causal Effect Moderation in Mobile Health. *Journal of the American Statistical Association* **113**, 1112–1121 (2018).
2. Khan, W. A. A., Jackson, M. L., Kennedy, G. A. & Conduit, R. A field investigation of the relationship between rotating shifts, sleep, mental health and physical activity of Australian paramedics. *Scientific Reports* **11**, 866 (2021).
3. Li, W., Yin, J., Cai, X., Cheng, X. & Wang, Y. Association between sleep duration and quality and depressive symptoms among university students: A cross-sectional study. *PLOS ONE* **15**, e0238811 (2020).
4. Bai, Y. *et al.* Comprehensive comparison of Apple Watch and Fitbit monitors in a free-living setting. *PLOS ONE* **16**, e0251975 (2021).
5. Fuller, D. *et al.* Reliability and Validity of Commercially Available Wearable Devices for Measuring Steps, Energy Expenditure, and Heart Rate: Systematic Review. *JMIR mHealth and uHealth* **8**, e18694 (2020).
